# Evaluation of a Novel Rapid Test System for the Detection of Specific IgE to Hymenoptera Venoms

**DOI:** 10.1155/2012/862023

**Published:** 2012-02-27

**Authors:** Nikolai Pfender, Ralf Lucassen, Nadine Offermann, Johannes Schulte-Pelkum, Margrit Fooke, Thilo Jakob

**Affiliations:** ^1^Allergy Research Group, Department of Dermatology, University Medical Center Freiburg, Hauptstraße 7, 79104 Freiburg, Germany; ^2^Dr. Fooke Laboratorien GmbH, Habichtweg 16, 41468 Neuss, Germany

## Abstract

*Background*. The Allergy Lateral Flow Assay (ALFA) is a novel rapid assay for the detection of sIgE to allergens. The objective of this study is the evaluation of ALFA for the detection of sIgE to bee venom (BV) and wasp venom (WV) in insect venom allergic patients. *Methods*. Specific IgE to BV and WV was analyzed by ALFA, ALLERG-O-LIQ, and ImmunoCAP in 80 insect venom allergic patients and 60 control sera. Sensitivity and specificity of ALFA and correlation of ALFA and ImmunoCAP results were calculated. *Results*. The sensitivity/specificity of ALFA to the diagnosis was 100%/83% for BV and 82%/97% for WV. For insect venom allergic patients, the Spearman correlation coefficient for ALFA versus ImmunoCAP was 0.79 for BV and 0.80 for WV. However, significant differences in the negative control groups were observed. *Conclusion*. ALFA represents a simple, robust, and reliable tool for the rapid detection of sIgE to insect venoms.

## 1. Introduction

Reliable diagnosis of insect venom allergy is based on the combination of patient's history, skin testing (SPT) and laboratory tests for the detection of specific IgE (sIgE) [1–4].

**Figure 1:**
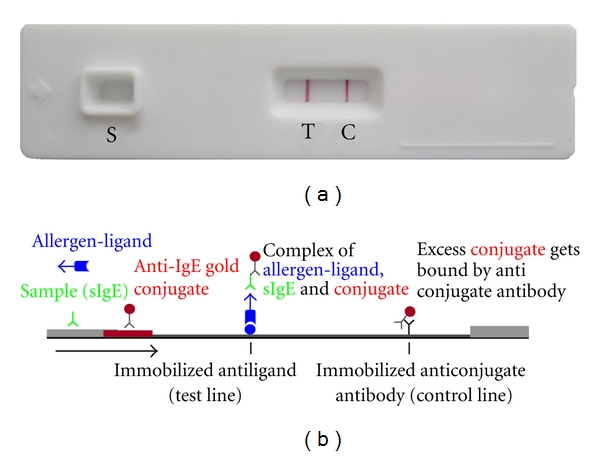
Principle of ALFA. A test cassette showing a positive test result is presented in (a) and the principle of the test in (b). The patient's sample is transferred to the sample application point. Immediately afterwards the allergen solution of interest is applied. During incubation time of 20 min, the liquid is driven through the device by capillary flow. Allergen specific IgE of the sample binds specifically to the corresponding antigens of the allergen solution. The antigens are labeled and are retained at the test line (T) by a capture molecule. At the same time the sIgE bound to the allergen is bound by an antibody coupled to colored particles (conjugate). The intensity of the color reaction at the test line is proportional to the amount of immune complexes consisting of ligand tagged antigens, sIgE, and IgE specific conjugate. The signal intensity ranges from faintly pink (low titer of sIgE) to dark ruby (high titer of sIgE). Access conjugate, which is not bound at the test line, will form a dark ruby control line (C) after 20 min of incubation.

The methodology for the measurement of sIgE has evolved during the recent years [5, 6]. Lately, rapid assays for the detection of sIgE as point-of-care diagnostics have been developed using various strategies [7, 8]. ALFA, the Allergy Lateral Flow Assay (Dr. Fooke Laboratorien GmbH, Neuss, Germany), combines the advantages of lateral flow devices with the flexibility of choosing different allergens as already shown for cat epithelia (e1),* Dermatophagoides pteronyssinus *(d1), *Alternaria alternata *(m6), birch pollen (t3) [9], and also in depth for timothy grass pollen (g6) [10]. In contrary to other rapid tests like the ImmunoCAP Rapid (Phadia, Uppsala, Sweden) [8], which employs a fixed panel of single allergens immobilized on membranes, ALFA utilizes liquid allergens and allergen mixtures. The open architecture of the ALFA system allows for the selection of the required allergen in combination with a universal basis set (see Figure 1). Biotinylated insect venoms of honey bee, *Apis mellifera* (BV) and wasp, *Vespula vulgaris* and *germanica* (WV) were characterized and prepared for use with ALFA. The objective of this study was the evaluation of ALFA, for the detection of sIgE to bee venom (BV) and wasp venom (WV) in a well-defined cohort of patients. Sera were also analyzed by ImmunoCAP (Phadia, Uppsala, Sweden) and ALLERG-O-LIQ (Dr. Fooke Laboratorien GmbH). The correlation between methods was calculated, and agreement between ALFA and the diagnosis (based on patient's history, positive skin test, and detection of sIgE by ImmunoCAP (≥0.35 kU/L)) was determined.

## 2. Patients and Methods

Sera of four groups were analyzed as follows.

(*A*) 40 patients with insect venom allergy to either bee (*n* = 12) or wasp (*n* = 28) venom with agreement of patient's history, sIgE against the given venom by ImmunoCAP and skin test. Mean age 41.8 y (SD 14.96 y). 23 (57.5%) males, 17 (42.5%) females. 3 (7.5%) Patients presented with anaphylactic reactions grade 1 (according to Ring and Messmer [11, 12]), 23 (57.5%) with anaphylactic reaction grade 2 and 14 (35%) with anaphylactic reaction grade 3 to insect venom sting. None of the patients presented with a grade 4 anaphylaxis. None of the patients were under immunosuppressive medication or specific immunotherapy. Mean total IgE was 78.7 kU/L (range 11.6–372 kU/L).
(*B*) 40 patients with insect venom allergy and double sensitization (serological) to bee and wasp venom, determined by ImmunoCAP, patient's history and skin test. Mean age 42.25 y (SD 17.5 y). 20 (50%) males, 20 (50%) females. 4 (10%) Patients presented with anaphylactic reactions grade 1 (according to Ring and Messmer [11, 12]), 27 (67.5%) with anaphylactic reaction grade 2 and 9 (22.5%) with anaphylactic reaction grade 3 to insect venom sting. None of the patients presented with a grade 4 anaphylaxis. None of the patients were under immunosuppressive medication or specific immunotherapy. Mean total IgE was 150.5 kU/L (range 16.5–667 kU/L).
(*C*) Atopic individuals (Sx1 positive, total IgE mean = 2986 kU/L, range 186–23813 kU/L, history of at least one atopic disease) without history of insect venom allergy (*n* = 30). Mean age 33.5 y (SD 15.75 y). 16 (53.34%) males, 14 (46.67%) females. 19 (63.34%) patients presented with atopic dermatitis, 12 (40%) patients with allergic rhinoconjunctivitis, and 7 (23.34%) with allergic asthma (some patients presented with two or three manifestations). None of the patients were under immunosuppressive medication or specific immunotherapy.
(*D*) Non atopic individuals without history of insect venom allergy (*n* = 30). None of the patients were under immunosuppressive medication or specific immunotherapy. Mean total IgE was 35.60 kU/L (range 2–99.3 kU/L).

Diagnosis of insect venom allergy was based on patient's history (anaphylaxis due to bee or wasp sting [11, 12]), positive skin test, and detection of sIgE by ImmunoCAP (≥0.35 kU/L).

### 2.1. Skin Test

In patients with insect venom allergy, skin prick tests with increasing concentrations of BV and WV (1, 10, 100 *μ*g/mL) (ALK- Abello, Reinbeck, Germany) were performed. If negative, intradermal tests at 1 *μ*g/mL were added as recommended by the position paper of the EAACI Interest Group on Insect Venom Hypersensitivity [13, 14]. Histamine chloride at a concentration of 1 mg/mL served as positive and saline solution (0.9%) as negative control.

### 2.2. Detection of sIgE

All sera in this study (*n* = 140) were assayed for sIgE to BV and WV by ALFA, ALLERG-O-LIQ, a reversed-type, quantitative, WHO 75/502 calibrated immunoassay [1], and ImmunoCAP according to the instructions given by the manufacturer. Specific IgE values >100 IU/mL were considered as 100 IU/mL. ALFA results were quantified by a scanning device with an appropriate software [10], which measures the colour intensity of the test line and evaluates the validity of the test run by measuring the existence and intensity of the control line. All samples were measured after the same incubation time of 20 min. The test values are converted into relative units (RUs). Using individually diluted allergen solutions, the test kits are validated for identical discrimination of positive and negative results. Cut-off values were set using ROC-decision analysis made with Analyze-it v2.21 for Excel (data not shown).

### 2.3. Statistical Analysis

Spearman correlation, *P*-values (1-tailed Student's *t*-test), and Receiver Operating Characteristic analysis were performed with GraphPad Prism Ver. 5.01.

## 3. Results and Discussion

### 3.1. Detection of sIgE to Bee and Wasp Venom

When compared to the diagnosis (based on patients history, skin test, and sIgE detection by ImmunoCAP) comparative receiver operating characteristic analysis of groups A and B revealed area under the curve values for ALFA of 0.97 (BV) and 0.91 (WV).

**Figure 2:**
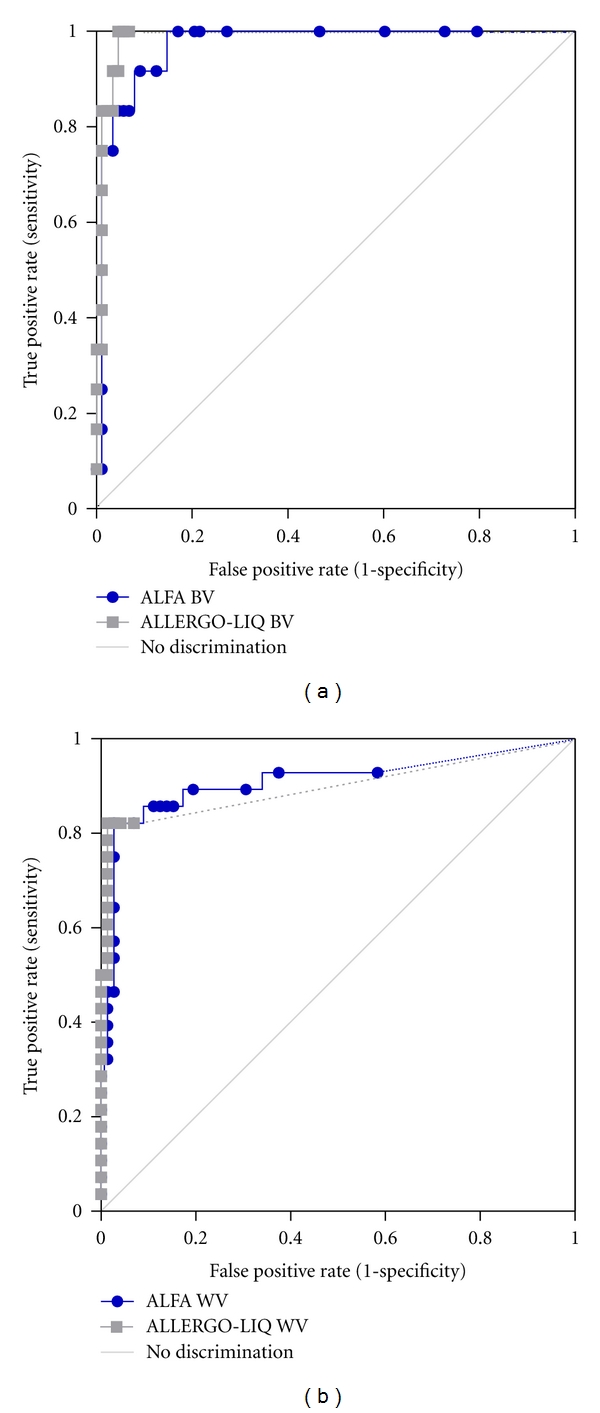
Receiver operating characteristic of ALFA and ALLERGO-LIQ for the diagnosis of bee (a) and wasp (b) venom allergy in monosensitized patients (group A) and control groups (C and D). Curve with dots indicates results for ALFA and curve with squares for ALLERGO-LIQ. Diagnosis of insect venom allergy was based on patient's history, skin testing, and detection of sIgE to bee or wasp venom by ImmunoCAP. Sensitivity/specificity for ALFA is 100%/83% (BV) and 82%/97% (WV) at a cut-off value of 10.0 RU and 100%/93% (BV) and 82%/93% (WV) for ALLERG-O-LIQ.

In monosensitized patients (group A only), 12/12 BV allergic patients and 23/28 WV allergic patients were positive in ALFA using a cut-off value for ALFA of 10.0 RU, corresponding to a sensitivity of 100% for BV and 82% for WV. Similar results were obtained by ALLERG-O-LIQ. In ALLERG-O-LIQ 12/12 BV allergic patients and 24/28 WV allergic patients were found positive (Figure 2).

For groups A and B high agreement between ALFA, ALLERG-O-LIQ, and skin tests of up to 92% was observed (data not shown). Using atopic (without history of insect venom allergy—group C) and nonatopic (group D) sera as controls, the specificity of ALFA for the detection of sIgE to BV was calculated as 83% and for sIgE to WV as 97% (Figure 2).

### 3.2. Comparison of ALFA and ImmunoCAP

**Figure 3:**
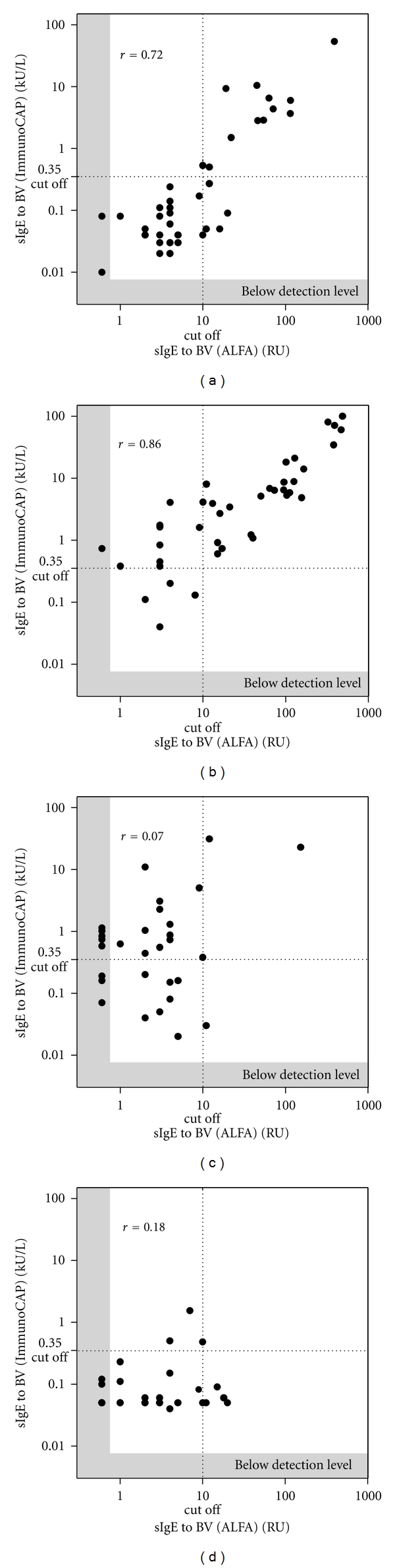
Spearman correlation diagram of ALFA versus ImmunoCAP for the detection of sIgE to bee venom. Quantitative agreement was found with a Spearman correlation coefficient of 0.72 (*P* < 0.0001) for group A (a), 0.86 (*P* < 0.0001) for group B (b), 0.07 (*P* = 0.37) for group C (c), and of 0.18 (*P* = 0.17) for group D (d) for the detection of sIgE to BV.

**Figure 4:**
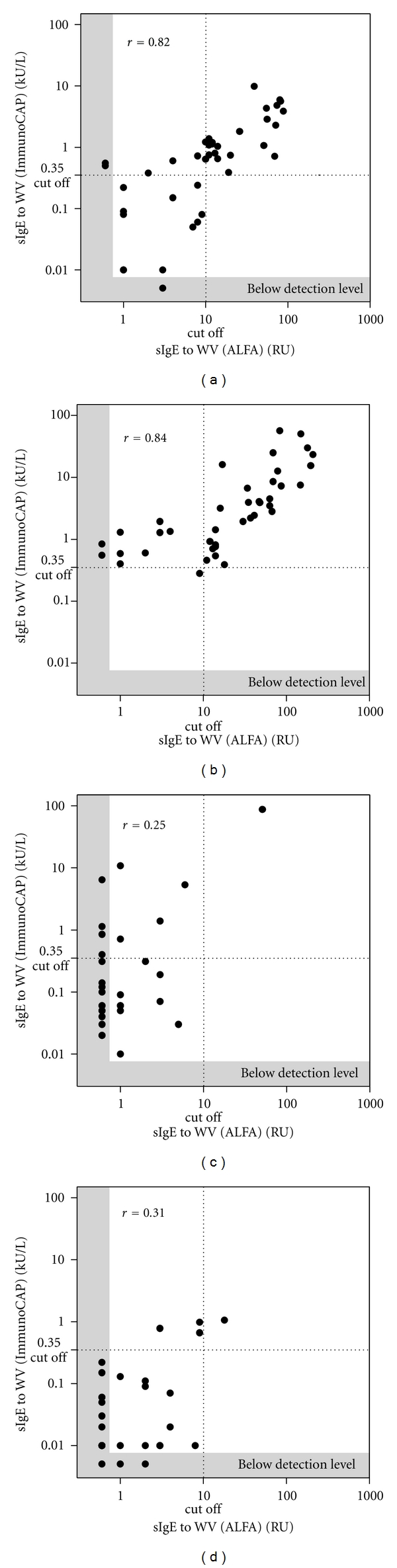
Spearman correlation diagram of ALFA versus ImmunoCAP for the detection of sIgE to wasp venom. Quantitative agreement was found with a Spearman correlation coefficient of 0.82 (*P* < 0.0001) for group A (a), 0.84 (*P* < 0.0001) for group B (b), 0.25 (*P* = 0.088) for group C (c), and of 0.31 (*P* = 0.047) for group D (d) for the detection of sIgE to WV.

For insect venom allergic patients (groups A and B) the Spearman correlation coefficient for ALFA versus ImmunoCAP was 0.79 (*P* < 0.0001) for BV and 0.80 (*P* < 0.0001) for WV (data not shown). Correlation of ALFA to ImmunoCAP for each group revealed a more defined picture. For the detection of sIgE to BV the Spearman correlation coefficient for ALFA versus ImmunoCAP was 0.72 (*P* < 0.0001) for group A, 0.86 (*P* < 0.0001) for group B, 0.07 (ns) for group C, and 0.18 (ns) for group D (Figure 3). For the detection of sIgE to WV the Spearman correlation coefficient for ALFA versus ImmunoCAP was 0.82 (*P* < 0.0001) for group A, 0.84 (*P* < 0.0001) for group B, 0.25 (ns) for group C, and 0.31 (*P* < 0.05) for group D (Figure 4).

**Figure 5:**
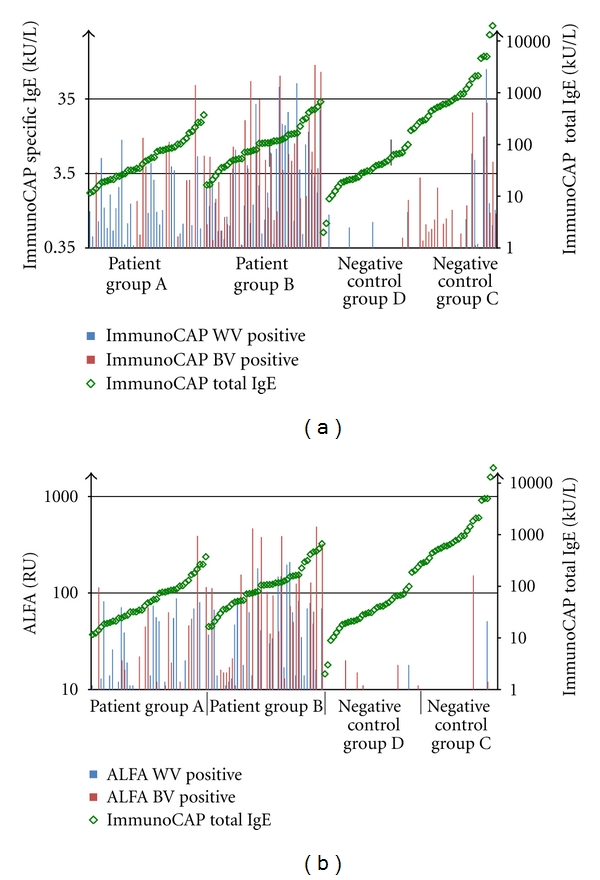
Graphical illustration of the relation of total IgE (green diamond, determined by ImmunoCAP) and sIgE to BV (red) and WV (blue) for ImmunoCAP (a) and ALFA (b) for each study group.

These results demonstrate a good quantitative agreement for the detection of sIgE to BV and WV between ALFA and ImmunoCAP for sera of allergic patients (groups A and B) but poor quantitative agreement for the control groups, especially for BV (groups C and D). This seems to be caused by a surprisingly high number of positive ImmunoCAP results in the control groups, in particular in the group of atopic individuals (without history of insect venom allergy) with high total IgE-a finding that has been described before for different allergens [15, 16]. In the present study in group C (atopic individuals with high total IgE) 19/30 (63.3%) of the samples were BV positive by ImmunoCAP compared to 4/30 (13.3%) samples tested positive by ALFA. For WV 9/30 (30.0%) samples tested positive by ImmunoCAP and 1/30 sample (3.3%) by ALFA. In group D positive BV results were observed for 3/30 sera (10%) by ImmunoCAP and 6/30 (20.0%) samples tested positive using ALFA. For WV the results in group D showed 4/30 (13.3%) positive tests by ImmunoCAP and 1/30 (3.3%) positive samples for ALFA, respectively, (Table 1). The different results of ALFA and ImmunoCAP in relation to total IgE level of the patient are shown in Figure 5.

Since sIgE to CCDs (cross-reactive carbohydrate determinants) can be responsible for major cross-reactivity of glycosylated allergens (e.g., of pollen, foods, insect venom, etc.), sIgE to CCDs could also account for the high rate of BV ImmunoCAP positives in the atopic negative controls (Group C). However analyzing CCD reactivity by ImmunoCAP showed that only less than 50% of the ImmunoCAP BV positive sera also displayed CCD reactivity (data not shown). This suggests that the high rate of BV positive results by ImmunoCAP in atopic patients (without history of insect venom allergy) with strongly elevated total IgE levels (mean = 2986 kU/L, range 186–23813 kU/L) cannot be explained by CCD reactivity but rather may reflect nonspecific IgE binding due to the solid phase architecture of the ImmunoCAP assay system.

Recently recombinant allergens have been introduced into the in vitro diagnostic of insect venom allergy. In particular for dissecting true double sensitization from cross-reactivity recombinant allergens seems to be of great relevance [17–19]. Since ALFA's open architecture allows for the use of any biotinylated allergen, the introduction of recombinant bee and wasp venom allergens will be useful and will certainly further increase the clinical sensitivity and specificity.

## 4. Conclusion

Detection of sIgE to bee and wasp venom by ALFA shows in the present study a good discrimination between sera of bee or wasp allergic patients and control sera with a comparable performance to the ALLERG-O-LIQ and ImmunoCAP system. Noteworthy ALFA detected no sIgE to BV in WV allergic patients and vice versa. In case of specificity, a high degree of correlation was found for ALFA and ALLERG-O-LIQ, whereas differing results were obtained using the ImmunoCAP system.

We conclude that the Allergy Lateral Flow assay together with the novel scanner-based system represents a versatile and reliable tool for the measurement of sIgE to insect venom allergens meeting the growing demand for digital documentation of laboratory results. Further studies with more allergens (e.g., recombinant insect venom allergens) are mandatory to further proof the applicability of the ALFA test system.

## Figures and Tables

**Table 1:** Results for the detection of bee and wasp venom of ALFA and ImmunoCAP for group A (*n* = 40), group B (*n* = 40), group C (*n* = 30), and group D (*n* = 30).

								
	ImmunoCAP				ALFA (cut-off 10 RU)		
	Bee venom positive	Bee venom negative	Wasp venom positive	Wasp venom negative	Bee venom positive	Bee venom negative	Wasp venom positive	Wasp venom negative
Group A	12	28	28	12	17	23	23	17
Group B	36	4	39	1	27	13	30	10
Group C	19	11	9	21	4	26	1	29
Group D	3	27	4	26	6	24	1	29
